# The number of remaining teeth as a risk indicator of cognitive impairment: A cross‐sectional clinical study in Sado Island

**DOI:** 10.1002/cre2.147

**Published:** 2018-11-30

**Authors:** Ayumi Kuroki, Noriko Sugita, Shigeki Komatsu, Minako Wakasugi, Akio Yokoseki, Akihiro Yoshihara, Tetsuo Kobayashi, Kazutoshi Nakamura, Takeshi Momotsu, Naoto Endo, Kenji Sato, Ichiei Narita, Hiromasa Yoshie

**Affiliations:** ^1^ Division of Periodontology, Department of Oral Biological Science Niigata University Graduate School of Medical and Dental Sciences Niigata Japan; ^2^ Sado General Hospital Niigata Japan; ^3^ Division of Comprehensive Geriatrics in Community Niigata University Graduate School of Medical and Dental Sciences Niigata Japan; ^4^ Department of Molecular Neuroscience, Resource Branch for Brain Disease Research, Brain Research Institute University of Niigata Niigata Japan; ^5^ Department of Oral Health and Welfare Niigata University Graduate School of Medical and Dental Sciences Niigata Japan; ^6^ General Dentistry and Clinical Education Unit Niigata University Medical and Dental Hospital Niigata Japan; ^7^ Division of Preventive Medicine Niigata University Graduate School of Medical and Dental Sciences Niigata Japan; ^8^ Division of Orthopedic Surgery, Department of Regenerative and Transplant Medicine Niigata University Graduate School of Medical and Dental Sciences Niigata Japan; ^9^ Division of Clinical Nephrology and Rheumatology Niigata University Graduate School of Medical and Dental Sciences Niigata Japan

**Keywords:** cognitive impairment, cross‐sectional, Mini‐Mental State Examination, remaining teeth

## Abstract

Most studies that have demonstrated an association between number of remaining teeth and cognitive impairment have treated teeth as a continuous variable, although the relationship is nonlinear. The aim of this cross‐sectional study was to determine the critical number of remaining teeth in hospital outpatients at which the association with cognitive impairment becomes apparent. Japanese adults living on Sado Island who visited Sado General Hospital were invited to participate in Project in Sado for Total Health. In total, 2,530 adults were interviewed and had their teeth counted; 1,476 of these individuals also completed the Mini‐Mental State Examination (MMSE) and underwent measurement of their serum high‐sensitivity C‐reactive protein (hsCRP) levels. Patients on dialysis and those with hsCRP ≥ 10 mg/L were excluded. The final study group consisted of 565 adults (290 men and 275 women) of mean age 69.8 (range 29–91) years. An MMSE score < 24 was considered to indicate cognitive impairment. The subjects were categorized according to whether they had an edentulous jaw or one to 10, 11–20, 21–27, or ≥28 remaining teeth. One hundred twenty‐eight of the 565 study participants were diagnosed to have cognitive impairment. Multiple logistic regression analysis revealed associations of cognitive impairment with older age, ischemic heart disease, smoking, and alcohol consumption. After adjustment for covariates, having one to 10 remaining teeth was significantly associated with cognitive impairment. There is a significant association between having only one to 10 remaining teeth and cognitive impairment in hospital outpatients.

## 1 INTRODUCTION

Japan has a superaging society, and growing numbers of patients with cognitive impairment have become an important health issue. A number of studies have attempted to identify modifiable risk factors for cognitive impairment. Considerable research attention has been focused on the association between cognitive impairment and number of remaining teeth (Stein, Desrosiers, Donegan, Yepes, & Kryscio, 2007). One possible explanation for this association is the prevalence of periodontitis, a common inflammatory disease caused by oral microorganisms and a major cause of tooth loss in adults (Pihlstrom, Michalowicz, & Johnson, 2005). Poor oral hygiene can lead to periodontitis and other oral diseases, such as dental caries and apical periodontitis (Kinane, Stathopoulou, & Papapanou, 2017; Page & Schroeder, 1976). Individuals with dementia are more likely to have poor oral health (Avlund, Holm‐Pedersen, Morse, Viitanen, & Winblad, 2004) and to lose more teeth than are those without dementia (Syrjälä et al., 2012). Further, patients with Alzheimer's disease and those on medication to reduce the risk of the sequelae of stroke tend to develop xerostomia, which is a risk factor for dental caries and periodontitis (Ship, DeCarli, Friedland, & Baum, 1990; Ship, Pillemer, & Baum, 2002). Moreover, several previous reports suggest that periodontitis might be a risk factor for cognitive impairment. Heneka et al. (2013) reported that inflammasomes have an important role in mild cognitive impairment and Alzheimer's disease in humans and confirmed this observation in experiments in mice. The inflammatory cytokines produced in periodontitis may enter the bloodstream and penetrate across the blood–brain barrier, and they induce production of β‐amyloid and tau phosphorylation (Gaur & Agnihotri, 2015).

Another view is that the stimulatory effect of chewing may help to prevent cognitive impairment. A previous study showed that sugarless chewing gum facilitated cognitive function, especially when glucose is coadministered (Stephens & Tunney, 2004). The association between cognitive impairment and number of remaining teeth is debatable from multiple viewpoints.

Most of the previous studies that assessed the association between the number of remaining teeth and cognitive impairment have treated the number of teeth as a continuous variable including edentulous individuals in statistical analysis (Akifusa et al., 2005; Saito et al., 2013). However, the relationship between the number of remaining teeth and cognitive function is nonlinear. Moreover, an edentulous jaw is a specific condition, so individuals with an edentulous jaw should be analyzed independently of those with remaining teeth.

The aim of this study was to determine the association between cognitive impairment and the number of remaining teeth in hospital outpatients.

## 2 MATERIAL AND METHODS

### 2.1 Participants

**Figure 1:**
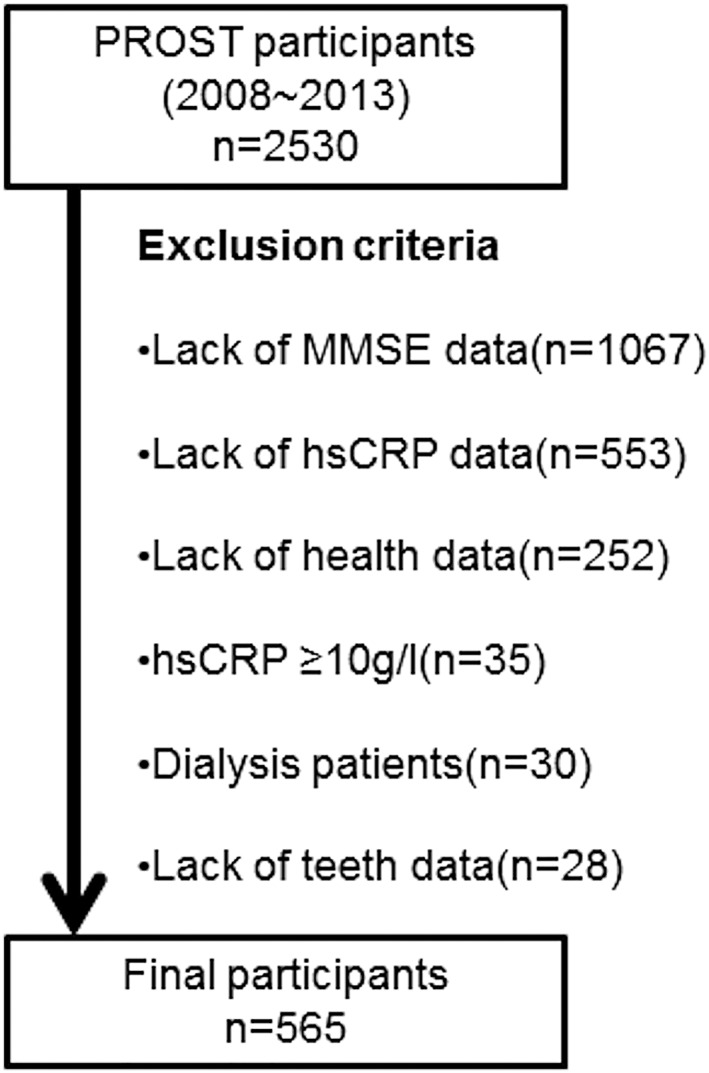
Selection of study participants. hsCRP, high‐sensitivity C‐reactive protein; MMSE, Mini‐Mental State Examination; PROST, Project in Sado for Total Health

All outpatients of Sado General Hospital were invited to participate in PROST (Project in Sado for Total Health), a hospital‐based cohort study that started in 2008 on Sado Island, Niigata Prefecture, Japan. The PROST registry was developed in collaboration with the Center for Inter‐Organ Communication Research at the Niigata University Graduate School of Medical and Dental Sciences. In total, 2,530 individuals aged 21–102 years had joined the PROST by 2013; all were interviewed and underwent counting of their remaining teeth between 2008 and 2013. Each subject's medical history was obtained from the clinical records. Mini‐Mental State Examination (MMSE) scores and serum high‐sensitivity C‐reactive protein (hsCRP) levels were measured in 1,476 of the 2,530 study participants. An MMSE score < 24 was considered to indicate cognitive impairment (Ideno, Takayama, Hayashi, Takagi, & Sugai, 2012; Pezzotti, Scalmana, Mastromattei, Di Lallo, & Group, 2008). Individuals with an hsCRP level ≥ 10 mg/L were considered to have acute inflammation and so were excluded (Roberts, Centers for Disease Control and Prevention, & American Heart Association, 2004), as were patients on dialysis (Figure 1).

Five hundred sixty‐five subjects were finally included in the study. The study protocol was approved by the medical ethics committee at Niigata University (Approval Number 511). Informed consent was obtained from all study participants. In case it was difficult for them to sign by themselves, their deputies signed.

### 2.2 Counting the number of teeth

The numbers of remaining teeth were counted by trained technicians. Wisdom teeth and residual roots with caps were included. The study participants were classified into five groups according to whether the number of remaining teeth was zero, one to 10, 11–20, 21–27, or ≥28 (Inomata et al., 2014).

### 2.3 Covariates

Age, sex, hsCRP level, body mass index (BMI), history of stroke, ischemic heart disease, hypertension, diabetes, oral health‐related variables (tooth brushing, history of gum swelling, and professional tooth cleaning), smoking and alcohol consumption, and dietary characteristics (intake of vegetables, fruit, and green tea) were considered as covariates in view of previous reports on risk factors for cognitive impairment (Bleckwenn et al., 2017; Cukierman, Gerstein, & Williamson, 2005; Elias, Elias, Sullivan, Wolf, & D'Agostino, 2003; Kitamura et al., 2016; Paganini‐Hill, White, & Atchison, 2012; Panza et al., 2012; Park, Park, Jun, Choi, & Suh, 2013; Patel, Coshall, Rudd, & Wolfe, 2002; Swan & Lessov‐Schlaggar, 2007; Watanabe et al., 2016). Age, sex, BMI, and blood pressure (BP) were recorded as part of the PROST registration procedure. BP was measured twice; average systolic BP ≥ 140 mmHg, average diastolic BP ≥ 90 mmHg, or use of antihypertensive medication was defined as hypertension. The medical history of each study participant was obtained from the clinical records. Data on oral health, habits, and dietary composition were collected using a self‐administered questionnaire.

### 2.4 Statistical analysis

The characteristics of the participants were evaluated according to sex using the Mann–Whitney *U* and the chi‐square test. Next, we evaluated the variables potentially associated with cognitive impairment in univariate analysis. The association between cognitive impairment and number of remaining teeth was then assessed in a multiple logistic regression model. Confounding factors were identified from the results of the univariate analysis. All statistical analyses were performed using SPSS Statistics for Windows, version 23.0 (IBM Corp., Armonk, NY).

## 3 RESULTS

One hundred twenty‐eight of the 565 participants in this study were diagnosed to have cognitive impairment.

The characteristics of the study participants are shown according to sex in Table 1. There were significant differences in rates of stroke, ischemic heart disease, diabetes, smoking, alcohol consumption, and vegetable consumption between men and women. The cutoff value for hsCRP was the median serum CRP concentration reported in a Japanese cohort study (Arima et al., 2008), and for BMI was 25.

**Table 1:** Characteristics of participants according to sex

Characteristic	Sex		*P* value
	Male *N* = 290	Female *N* = 275	
MMSE < 24, *n* (%)	62 (21.4)	66 (24.0)	0.46
Number of teeth, *n* (%)			
0	37 (12.8)	41 (14.9)	0.19
1–10	57 (19.7)	48 (17.5)	
11–20	49 (16.9)	64 (23.3)	
21–27	101 (34.8)	76 (27.6)	
≥28	46 (15.9)	46 (16.7)	
Age (year)^a^	71 (62, 77)	73 (65, 79)	0.06
hsCRP ≥ 0.4 mg/L, *n* (%)	159 (54.8)	129 (46.9)	0.60
BMI ≥ 25, *n* (%)	114 (39.3)	88 (32.0)	0.70
Stroke, *n* (%)	48 (16.6)	30 (10.9)	0.05
Ischemic heart disease, *n* (%)	27 (9.3)	11 (4.0)	0.01
Hypertension, *n* (%)	209 (72.1)	187 (68.0)	0.29
Diabetes, *n* (%)	110 (37.9)	76 (27.6)	0.01
1 time or more brush teeth in a day, *n* (%)	276 (95.2)	269 (97.8)	0.09
History of gum swelling, *n* (%)	96 (33.1)	97 (35.3)	0.59
Receive professional tooth cleaning regularly, *n* (%)	59 (20.3)	73 (26.5)	0.08
Have smoking habit, *n* (%)	218 (75.2)	30 (10.9)	<0.001
Drinking habit, *n* (%)			
Chance drinker	95 (32.8)	70 (25.5)	<0.001
Drinker at least once/week	133 (45.9)	32 (11.6)	
Consumption of vegetable every day, *n* (%)	249 (85.9)	258 (93.8)	0.02
Consumption of fruit every day, *n* (%)	145 (50.0)	161 (58.5)	0.04
Consumption of green tea every day, *n* (%)	113 (39.0)	111 (40.4)	0.73

*Note*. MMSE: Mini‐Mental State Examination; hsCRP: high‐sensitivity C‐reactive protein; BMI: body mass index.

a The data are presented as the number (percentage) or the median (25th and 75th percentiles) as appropriate. Mann–Whitney *U* tests or chi‐square tests were used. The cutoff value for hsCRP is the median serum CRP concentration reported for the Japanese population (Arima et al., 2008).

Table 2 shows the odds ratios for the candidate variables for cognitive impairment adjusted for sex and age. To clarify more details on the associations, we use quartiles for hsCRP and BMI.

**Table 2:** Odds ratios for cognitive impairment (MMSE < 24) adjusted for age and sex (*N* = 565)

Predictor variable	*P* value	Adjusted odds ratio	95% CI
Age*	<0.001	1.09	[1.06, 1.12]
Sex (male)	0.91	0.98	[0.64, .48]
hsCRP			
1st quartile		Reference	
2nd quartile	0.67	0.87	[0.47, 1.62]
3rd quartile	0.43	1.27	[0.70, 2.29]
4th quartile	0.11	1.61	[0.90, 2.58]
BMI			
1st quartile		Reference	
2nd quartile	0.42	0.79	[0.45, 1.35]
3rd quartile	0.07	0.57	[0.31, 1.04]
4th quartile	0.34	0.76	[0.45, 1.39]
Number of teeth*			
0	0.06	2.38	[0.95, 5.93]
1–10	0.03	3.75	[1.57, 8.93]
11–20	0.39	1.48	[0.60, 3.62]
21–27	0.61	1.26	[0.53, 2.99]
≥28		Reference	
Stroke	0.07	1.65	[0.97, 2.83]
Ischemic heart disease*	0.001	3.28	[1.61, 6.70]
Hypertension	0.27	1.31	[0.81, 2.10]
Diabetes	0.99	1.00	[0.64, 1.55]
1 time or more brush teeth in a day	0.42	1.53	[0.55, 4.24]
History of gum swelling, *n* (%)	0.06	0.64	[0.40, 1.01]
Receive professional tooth cleaning regularly, *n* (%)	0.10	0.64	[0.38, 1.08]
Have smoking habit*	0.01	2.25	[1.21, 4.17]
Drinking habit*			
None		Reference	
Chance drinker	0.09	0.60	[0.33, 1.08]
1 time at least/week	0.04	0.56	[0.33, 0.96]
Vegetable consumption	0.71	1.17	[0.50, 2.75]
Fruit consumption	0.34	1.23	[0.80, 1.88]
Green tea consumption	0.09	1.45	[0.94, 2.24]

*Note*. Sex was adjusted for age, and age was adjusted for sex. BMI: body mass index; CI: confidence interval; hsCRP: high‐sensitivity C‐reactive protein; MMSE: Mini‐Mental State Examination; OR: odds ratio.

* *P* < 0.05, logistic regression analysis.

We identified age, number of remaining teeth, ischemic heart disease, smoking, and alcohol as confounding factors and included them in the subsequent multiple logistic regression analysis.

As shown in Table 3, cognitive impairment was associated with older age, one to 10 remaining teeth, and a history of ischemic heart disease, smoking, and alcohol consumption.

**Table 3:** Multiple logistic regression analysis for cognitive impairment (MMSE score < 24) as an outcome

Variable	*P* value	Adjusted odds ratio	95% CI
Age	<0.001	1.07	[1.04, 1.10]
Number of teeth			
0	0.13	2.07	[0.82, 5.23]
1–10	0.01	3.29	[1.36, 7.96]
11–20	0.51	1.36	[0.55, 3.36]
21–27	0.80	1.12	[0.47, 2.72]
≥28		Reference	
Ischemic heart disease	0.01	2.73	[1.32, 5.67]
Have smoking habit	0.04	1.68	[1.02, 2.78]
Drinking habit			
None		Reference	
Chance drinker	0.05	0.55	[0.30, 1.00]
1 time at least/week	0.01	0.49	[0.28, 0.86]

*Note*. Each variable was adjusted for all other variables. CI: confidence interval; MMSE: Mini‐Mental State Examination; OR: odds ratio.

## 4 DISCUSSION

In this study, the association with cognitive impairment was stronger in the group with one to 10 remaining teeth than in the edentulous group. This result contributes to a better understanding of the previously reported association between the number of remaining teeth and cognitive impairment. A possible explanation for this finding might be the difference in the stability of mastication. Occlusal disharmony is a chronic stress and thereby enhances the secretion of corticosteroids or other stress‐activated neuronal responses, which may trigger cognitive impairments, especially in the elderly (Karlamangla, Singer, Chodosh, McEwen, & Seeman, 2005; Seeman, McEwen, Singer, Albert, & Rowe, 1997). Further, in another study, wearers of partial dentures tended to have lower oral health‐related quality of life because adaptation to wearing their dentures was more difficult than in wearers of complete dentures (Bae, Kim, Paik, & Kim, 2006). Another possible explanation is the decreased opportunities to visit a dentist. As older adults with dementia are likely to have less frequent dental visit than are individuals with normal cognitive function (Lee, Wu, & Plassman, 2015), the last remaining teeth indicated for extraction may survive longer in their mouths. Further investigations, including detailed assessment of oral health status and history of dental treatment in all study participants, would be needed.

Serum hsCRP is a marker of the systemic response to inflammation. A previous PROST study reported an association between a higher CRP concentration and lower cognitive function, suggesting that chronic inflammation may have an effect on cognitive status (Watanabe et al., 2016). Periodontitis has been reported to increase the serum hsCRP level (Nakajima et al., 2010). We expected that elevated CRP would be the link between the number of remaining teeth and cognitive status. However, the association between CRP levels and cognitive status was not statistically significant in this study. Although the association was stronger in women in the previous study (Watanabe et al., 2016), we could not test the association in women because of insufficient numbers. No significant differences in serum hsCRP levels were found between the groups with different numbers of remaining teeth.

In this study, we found that a history of ischemic heart disease and smoking were associated with cognitive impairment, which is consistent with previous studies (Bleckwenn et al., 2017; Park et al., 2013). Ischemic heart disease was the main cause of heart failure, and posterior cortical areas of the brain may be particularly vulnerable to the decrease in brain perfusion associated with heart failure, suggesting that functional deficits in these regions might be relevant to the pathophysiology of cognitive impairment in patients with heart failure (Alves et al., 2005). Smoking has been reported to affect the risk of cognitive impairment and Alzheimer's disease because of its harmful effects in terms of oxidative stress and triggering inflammatory and atherosclerotic processes (Swan & Lessov‐Schlaggar, 2007).

Although high BMI, history of stroke, hypertension, and diabetes are known risk factors for cognitive impairment (Cukierman et al., 2005; Elias et al., 2003; Patel et al., 2002), these associations did not reach statistical significance in our study, possibly because of its relatively limited sample size. Further, the older age of our study participants may have attenuated the effects of these risk factors. A previous report suggested that obesity is a risk for cognitive impairment in middle‐aged individuals but not in the elderly (Shaw, Sachdev, Abhayaratna, Anstey, & Cherbuin, 2017).

The results of the questionnaires indicated no association of cognitive impairment with tooth brushing, swelling of the gums, or professional tooth cleaning. We could not obtain records for periodontal parameters, dental caries, or control of dental plaque, so we had to rely on self‐reported data for assessment of oral health. However, the concordance of these self‐reports with the actual severity of inflammation or oral hygiene status may be limited.

In public health, MMSE was sufficiently accurate in detecting patients with cognitive impairment, particularly in those with dementia (Pezzotti et al., 2008). MMSE cutoff score of 23 points indicates more than 90% sensitivity and specificity to identify cognitive function in Japanese adults (Ideno et al., 2012). However, the final diagnosis of cognitive impairment would require further assessments by a neurologist or geriatrician, including magnetic resonance imaging or computed tomography data.

Although the mechanism is unclear, light‐to‐moderate alcohol consumption has often been reported to be associated with a reduced risk of incident overall dementia and Alzheimer's disease (Panza et al., 2012). Our results may support this association, but we have no detailed data on actual alcohol consumption levels in our study participants. Moreover, it is difficult to identify and maintain a beneficial level of alcohol consumption while avoiding an excessive alcohol intake.

One of the limitations of this study is that we were unable to collect data on oral diseases such as periodontitis, on denture wearing, or on chewing ability. Further, we could not measure serum inflammatory mediators, including interleukin‐1, interleukin‐6, and tumor necrosis factor alpha, which have been reported to correlate with cognitive impairment (Gaur & Agnihotri, 2015). Next, we did not assess the subjects' education level and state of family living together, important factors associated with cognitive impairment (Crum, Anthony, Bassett, & Folstein, 1993; Webber, Fox, & Burnette, 1994). This may also have confounded the observed associations.

The number of participants who recorded high values of hsCRP or had dialysis treatment was too small for statistical analysis. However, it would be valuable to analyze how these factors correlate to both the MMSE score and the number of teeth in future research.

Finally, causative relationships could not be determined because of the cross‐sectional study design. Further studies in the PROST cohort are expected to clarify these issues.

## 5 CONCLUSION

There is a significant association between cognitive impairment and having only one to 10 remaining teeth in Japanese adults.

## CONFLICT OF INTEREST

The authors declare no conflict of interest.
